# Draft Genome Sequences of 113 Escherichia coli Strains Isolated from Intramammary Infections in Dairy Cattle

**DOI:** 10.1128/MRA.01464-20

**Published:** 2021-02-18

**Authors:** Dongyun Jung, Soyoun Park, Janina Ruffini, Simon Dufour, Jennifer Ronholm

**Affiliations:** a Faculty of Agricultural and Environmental Sciences, Macdonald Campus, McGill University, Sainte-Anne-de-Bellevue, Québec, Canada; b Département de Pathologie et Microbiologie, Faculté de Médecine Vétérinaire, Université de Montréal, Saint-Hyacinthe, Québec, Canada; c Regroupement FRQNT Op+Lait, Saint-Hyacinthe, Québec, Canada; d Canadian Bovine Mastitis Research Network, Saint-Hyacinthe, Québec, Canada; University of Maryland School of Medicine

## Abstract

Escherichia coli is one of the most common etiological agents responsible for clinical bovine mastitis. Here, we report the draft genome sequences and annotations of 113 E. coli strains that were isolated from Holstein cows with intramammary infections in Canada.

## ANNOUNCEMENT

Escherichia coli is a major pathogen that is responsible for clinical mastitis in dairy cows. Dairy cows with intramammary E. coli infections result in economic losses for the dairy industry due to reductions in milk yield, premature culling, and treatment costs (1). Bovine mastitis cases caused by E. coli have increased, relative to contagious mastitis, over the past few decades (2–5). However, the etiology of E. coli mastitis is not fully understood. Several attempts have been made to define a specific mammary pathogenic E. coli pathotype, but a set of core virulence factors has not yet been defined (6–10). Therefore, this sequencing project was conducted to sequence additional E. coli isolates from intramammary infections to better define the molecular characteristics and pangenomic composition of mammary pathogenic E. coli.

The Canadian Bovine Mastitis Research Network maintains a culture collection of mastitis isolates that were collected from 91 dairy farms across Canada over a 2-year period (2007 and 2008) (11). All strains sequenced in this project were obtained from this collection. Each isolate was collected from cows experiencing clinical mastitis, either on the day of diagnosis or during subsequent post-clinical mastitis follow-up sampling.

Here, we present the draft genome sequences of 113 E. coli isolates from bovine mastitis cases in Canada. Briefly, the isolates were cultured from raw milk samples plated on biplates containing a 1:1 mixture of Columbia agar with 5% sheep blood and MacConkey agar. Standard biochemical tests were performed to confirm that the isolates were E. coli (lactose and indole positive and oxidase and citrate negative) (11). To extract DNA for sequencing, E. coli was grown overnight on tryptic soy agar (TSA) and a well-isolated single colony was selected for DNA isolation. DNA was extracted using DNAzol reagent (Invitrogen, Carlsbad, CA) and the Maxwell RSC blood DNA kit (Promega, Madison, WI) following the manufacturer’s instructions. The short-read sequence data were generated by preparing paired-end libraries with the Nextera Flex DNA library preparation kit (Illumina, San Diego, CA) and Nextera DNA CD indexes (96 indexes and 96 samples), with sequencing on a MiSeq benchtop sequencer (Illumina) for 301 cycles in each direction. The reads were assembled *de novo* into high-quality draft genomes with ProkaryoteAssembly v0.1.6 (https://github.com/bfssi-forest-dussault/ProkaryoteAssembly). This pipeline consists of quality control and trimming of low-quality sequences (Q value, <20) using BBDuk (BBMap v38.79), error correction using Tadpole (BBMap v38.79), assembly using SKESA v2.4, alignment of error-corrected reads against the draft assembly (BBMap v38.79), and polishing of the assembly using Pilon v1.23 (12–14). Default parameters were used for all software with the exception of the trimming by BBDuk, for which the command trimq=20 2> {stats_out} was used. This assembly resulted in nonoverlapping contiguous sequences for each genome (Table 1). The gene predictions and annotations were performed by the National Center for Biotechnology Information (NCBI) through the Prokaryotic Genome Annotation Pipeline (PGAP) (15, 16).

**TABLE 1:** Sequencing and annotation results for E. coli strains isolated from bovine intramammary infections

Strain identification no.	SRA accession no.	GenBank accession no.	Minimum/maximum/mean read length (bp)	Total no. of reads	*N*_50_ (bp)	GC content (%)	Draft genome size (bp)	Coverage (×)	No. of contigs	No. of coding sequences	No. of RNAs
30800472	SRR11308090	JAASLI000000000	10/301/207.99	2,364,158	193,208	50.48	4,750,119	102.1338	50	4,387	87
32608571	SRR11308089	JAASLJ000000000	10/301/227.71	909,888	114,214	49.64	4,851,253	41.9501	90	4,536	81
40202761	SRR11308048	JAASLK000000000	10/301/223.17	3,878,274	111,816	50.5	5,187,220	163.3316	149	4,940	85
41100011	SRR11308037	JAASLL000000000	10/301/234.4	554,160	223,217	50.23	5,053,044	25.4401	48	4,657	82
11902638	SRR11308015	JAASLN000000000	10/301/242.74	3,657,858	103,836	50.69	4,737,985	184.1148	110	4,358	82
21002809	SRR11308004	JAASLO000000000	10/301/248.71	1,649,400	153,424	50.66	5,154,086	77.7807	104	4,936	80
10703014	SRR11307993	JAASLP000000000	10/301/245.13	5,932,068	122,333	50.63	5,141,782	274.8045	103	4,918	80
21003035	SRR11307982	JAASLQ000000000	10/301/243.34	1,231,714	179,599	50.6	4,754,917	62.1866	50	4,397	80
30415454	SRR11307971	JAASLR000000000	10/301/242.31	1,793,372	204,030	50.38	4,885,000	87.9337	54	4,557	83
20202040	SRR11308088	JAASLS000000000	10/301/233.07	631,896	93,614	50.14	4,932,918	29.5432	97	4,649	82
41300398	SRR11308077	JAASLT000000000	10/301/243.97	2,946,554	132,594	50.55	4,975,487	141.3809	116	4,699	81
41613979	SRR11308066	JAASLU000000000	10/301/222.99	2,422,532	111,686	50.39	5,135,635	103.6631	110	4,845	86
41514177	SRR11308055	JAASLV000000000	10/301/249.13	3,105,796	151,797	50.62	4,700,033	162.6354	60	4,428	84
21210525	SRR11308054	JAASLW000000000	10/301/213.23	2,257,290	330,509	49.88	5,003,111	93.7664	49	4,607	84
32708899	SRR11308053	JAASLX000000000	10/301/247.8	3,393,892	293,295	50.53	5,032,037	165.58	66	4,707	88
21012914	SRR11308052	JAASLY000000000	10/301/225.52	1,323,618	227,096	50.43	4,848,643	60.7037	51	4,489	86
10415566	SRR11308051	JAASLZ000000000	10/301/226.06	1,124,532	103,590	50.23	4,953,754	50.5923	92	4,727	83
40714004	SRR11308049	JAASMB000000000	10/301/251.77	1,313,622	154,432	50.4	4,994,627	65.5961	81	4,761	83
10715833	SRR11308047	JAASMC000000000	10/301/247.77	1,533,606	193,164	50.3	4,857,470	77.5656	41	4,552	80
41701140	SRR11308045	JAASME000000000	10/301/242.63	2,348,114	113,663	49.95	4,842,560	114.9075	101	4,524	84
20510930	SRR11308044	JAASMF000000000	10/301/199.56	746,156	108,945	50.32	5,069,658	28.9223	102	4,794	78
21413445	SRR11308043	JAASMG000000000	10/301/248.99	1,945,810	203,995	50.52	4,973,772	96.2797	68	4,709	86
31313964	SRR11308042	JAASMH000000000	10/301/203.03	1,159,260	210,167	50.44	4,994,978	46.5809	66	4,698	79
10213407	SRR11308041	JAASMI000000000	10/301/219.99	2,760,748	112,623	49.65	4,918,545	121.6872	88	4,678	84
21012853	SRR11308040	JAASMJ000000000	10/301/228.69	4,344,634	136,859	50.75	4,804,819	204.6487	66	4,484	81
31716253	SRR11308039	JAASMK000000000	10/301/205.73	1,577,086	111,943	50.45	4,748,664	67.3299	92	4,442	84
32016505	SRR11308038	JAASML000000000	10/301/225.68	2,108,832	236,090	50.53	4,609,704	102.185	32	4,237	82
41602577	SRR11308036	JAASMM000000000	10/301/199.4	1,389,514	111,698	50.48	5,041,359	53.8835	114	4,681	82
10117996	SRR11308035	JAASMN000000000	10/301/199.7	1,312,214	228,805	50.38	5,118,504	50.1807	63	4,763	84
32400021	SRR11308034	JAASMO000000000	10/301/211.29	5,799,496	192,460	50.58	5,124,294	235.433	76	4,820	80
30215009	SRR11308033	JAASMP000000000	10/301/207.93	811,486	132,211	50.08	5,158,714	32.2906	84	4,888	82
22113962	SRR11308032	JAASMQ000000000	10/301/233.72	1,884,730	132,522	50.31	4,696,397	92.7238	80	4,358	86
20314330	SRR11308031	JAASMR000000000	10/301/239.58	1,720,450	185,339	50.22	4,734,962	86.2987	49	4,470	80
11211990	SRR11308030	JAASMS000000000	10/301/222.23	735,520	239,890	50.07	4,892,505	33.2793	45	4,575	78
21317859	SRR11308029	JAASMT000000000	10/301/208.78	1,288,588	154,578	50.33	4,993,154	52.818	79	4,685	79
20108939	SRR11308028	JAASMU000000000	10/301/236.83	1,524,946	185,706	50.19	5,020,347	70.9977	69	4,791	83
21309335	SRR11308027	JAASMV000000000	10/301/227.75	2,260,410	193,073	50.1	4,952,614	102.6247	57	4,719	80
31209373	SRR11308025	JAASMW000000000	10/301/231.55	1,469,280	119,386	50.37	5,128,882	65.555	107	4,878	85
40611099	SRR11308024	JAASMX000000000	10/301/199.17	1,166,816	177,254	50.69	4,682,366	48.9991	51	4,346	81
21202100	SRR11308023	JAASMY000000000	10/301/232.19	1,636,822	268,024	50.34	4,981,971	75.3766	50	4,698	82
22406217	SRR11308021	JAASNA000000000	10/301/207.26	934,110	287,425	50.07	4,832,526	39.6709	37	4,508	83
11909132	SRR11308020	JAASNB000000000	10/301/227.22	2,197,572	99,251	50.58	4,749,854	102.9988	88	4,439	76
31709637	SRR11308019	JAASNC000000000	10/301/234.51	2,734,192	136,218	50.27	4,984,331	127.0759	79	4,723	87
10109199	SRR11308018	JAASND000000000	10/301/236.85	901,644	246,457	50.48	4,835,658	43.8138	40	4,471	83
10709818	SRR11308017	JAASNE000000000	10/301/234.19	1,804,446	154,007	50.35	4,870,029	85.8389	56	4,540	89
10208472	SRR11308014	JAASNG000000000	10/301/199.36	1,183,272	154,627	50.23	4,838,431	48.1355	58	4,519	83
32107654	SRR11308013	JAASNH000000000	10/301/225.1	1,173,454	124,636	50.18	4,531,629	57.3691	88	4,275	80
40706726	SRR11308011	JAASNJ000000000	10/301/250.53	585,814	166,923	50.55	4,717,991	30.7536	78	4,361	88
41207659	SRR11308010	JAASNK000000000	10/301/224.74	1,275,348	127,997	50.09	5,283,912	52.581	127	4,564	78
32009354	SRR11308009	JAASNL000000000	10/301/200.73	1,495,288	161,357	50.55	4,786,888	61.5108	68	4,476	74
21105562	SRR11308006	JAASNO000000000	10/301/210.17	592,924	186,563	50.76	4,849,769	25.4318	61	4,464	76
22306838	SRR11308005	JAASNP000000000	10/301/221.21	480,772	112,813	50.54	4,615,236	22.8586	86	4,296	79
30300071	SRR11308003	JAASNQ000000000	10/301/231.29	728,494	81,571	51.06	4,912,775	33.7192	125	4,564	78
11800057	SRR11308002	JAASNR000000000	10/301/133.81	978,798	108,945	50.51	4,962,279	25.8738	122	4,660	65
41505922	SRR11308001	JAASNS000000000	10/301/203.69	1,636,204	187,131	50.37	4,854,471	67.7096	55	4,543	79
11603481	SRR11308000	JAASNT000000000	10/301/190.52	1,073,206	154,416	50.37	4,906,285	41.2121	70	4,606	77
10700396	SRR11307999	JAASNU000000000	10/301/207.98	1,371,232	136,402	50.18	5,230,948	53.2558	96	4,969	83
10800294	SRR11307998	JAASNV000000000	10/301/231.64	670,740	312,323	50.47	5,021,526	30.5877	60	4,703	82
32608632	SRR11307997	JAASNW000000000	10/301/204.12	1,241,752	125,807	50.37	4,657,298	53.5676	93	4,313	80
22713162	SRR11307995	JAASNY000000000	10/301/225.33	1,600,488	126,545	50.41	4,526,770	79.1078	67	4,181	80
20814168	SRR11307994	JAASNZ000000000	10/301/235.33	2,223,726	124,094	50.48	4,887,593	105.3999	80	4,596	88
21211973	SRR11307992	JAASOA000000000	10/301/237.63	1,153,246	166,304	50.53	4,896,715	55.153	76	4,582	78
21212574	SRR11307991	JAASOB000000000	10/301/231.83	1,923,686	330,527	50.03	5,079,266	86.8967	46	4,680	87
41809617	SRR11307990	JAASOC000000000	10/301/226.96	2,024,622	200,222	50.5	4,859,115	93.5295	50	4,554	77
40717203	SRR11307989	JAASOD000000000	10/301/226.58	1,338,568	154,627	50.27	4,845,553	61.8463	64	4,529	83
40115481	SRR11307988	JAASOE000000000	10/301/244.21	2,269,900	212,440	50.41	4,870,786	112.8762	41	4,557	83
10417409	SRR11307986	JAASOG000000000	10/301/227.11	1,824,880	112,623	50.28	4,923,837	82.8244	86	4,683	84
40816739	SRR11307985	JAASOH000000000	10/301/226.31	4,715,758	233,858	50.39	4,809,083	217.3338	79	4,476	83
10216675	SRR11307984	JAASOI000000000	10/301/241.23	1,812,270	264,347	50.33	4,720,701	91.8694	45	4,421	82
21914256	SRR11307983	JAASOJ000000000	10/301/207.67	1,362,358	175,794	50.56	5,064,067	55.0172	100	4,789	87
21914232	SRR11307981	JAASOK000000000	10/301/227.55	1,326,020	126,629	50.35	4,821,420	61.5454	91	4,511	85
31716895	SRR11307980	JAASOL000000000	10/301/219.14	1,688,170	119,330	50.57	4,750,025	76.9743	90	4,446	85
10816417	SRR11307979	JAASOM000000000	10/301/241.06	7,240,116	123,143	50.01	4,799,871	360.2256	81	4,478	88
21017438	SRR11307978	JAASON000000000	10/301/199.98	1,675,104	295,209	50.35	4,859,493	68.2729	38	4,476	86
40317434	SRR11307977	JAASOO000000000	10/301/200.84	704,164	171,927	49.85	4,992,560	27.5833	69	4,608	78
21215100	SRR11307976	JAASOP000000000	10/301/132.17	792,686	69,303	50.31	4,614,490	22.335	132	4,297	53
21416415	SRR11307975	JAASOQ000000000	10/301/225.59	1,268,312	129,240	50.71	4,762,289	59.2865	79	4,435	84
20508456	SRR11307974	JAASOR000000000	10/301/225.44	2,045,884	131,795	50.2	4,922,560	92.6325	75	4,644	84
22710987	SRR11307973	JAASOS000000000	10/301/238.97	2,628,412	243,045	50.26	5,088,960	122.3065	40	4,792	81
22510686	SRR11307969	JAASOV000000000	10/301/205.31	1,276,116	86,398	50.17	5,062,593	50.6544	127	4,799	82
40614038	SRR11307968	JAASOW000000000	10/301/206.7	1,372,058	134,798	50.33	5,082,956	54.9489	82	4,814	85
30812765	SRR11307967	JAASOX000000000	10/301/201.24	1,175,542	86,861	50.11	5,059,376	45.7058	127	4,799	81
31412124	SRR11307966	JAASOY000000000	10/301/200.78	1,607,622	138,175	50.2	4,981,336	63.982	72	4,713	82
30810358	SRR11307965	JAASOZ000000000	10/301/203.53	731,670	77,317	49.99	5,072,442	28.848	134	4,810	79
41810132	SRR11307964	JAASPA000000000	10/301/200.47	1,575,764	115,436	50.42	5,361,537	57.9845	115	5,070	87
22114969	SRR11307963	JAASPB000000000	10/301/227.04	1,344,794	210,167	50.31	4,878,225	61.6622	51	4,547	81
21415616	SRR11307962	JAASPC000000000	10/301/189.08	1,148,816	117,077	50.61	4,873,504	44.1331	82	4,571	84
21013690	SRR11307961	JAASPD000000000	10/301/203.68	812,998	109,940	50.4	5,059,989	32.1857	132	4,780	84
21317521	SRR11308087	JAASPE000000000	10/301/228.03	5,034,608	515,670	50.35	5,153,657	220.5654	56	4,745	87
21314933	SRR11308086	JAASPF000000000	10/301/225.84	991,982	171,527	50.5	4,779,423	46.1718	64	4,473	80
21314773	SRR11308085	JAASPG000000000	10/301/226.59	1,885,696	119,489	50.49	4,890,208	86.72	78	4,251	81
22115768	SRR11308084	JAASPH000000000	10/301/203.65	1,299,512	155,324	50.15	4,665,474	56.18	56	4,345	83
21014604	SRR11308083	JAASPI000000000	10/301/208.03	2,650,938	139,784	50.42	4,977,882	109.0322	83	4,712	84
20316198	SRR11308082	JAASPJ000000000	10/301/135.49	981,532	160,999	50.42	4,939,636	26.38	112	4,603	69
20214326	SRR11308081	JAASPK000000000	10/301/244.62	639,356	172,536	50.64	4,683,212	33.0247	56	4,346	80
20216764	SRR11308080	JAASPL000000000	10/301/243.06	2,438,794	360,642	50.33	4,897,539	119.6603	28	4,505	87
40910260	SRR11308079	JAASPM000000000	10/301/226.59	1,885,696	303,620	50.49	4,890,208	86.72	34	4,518	83
40709628	SRR11308078	JAASPN000000000	10/301/224.71	935,720	119,115	50.75	4,740,552	44.022	105	4,412	81
21310034	SRR11308076	JAASPO000000000	10/301/226.36	1,076,372	156,656	50.77	4,671,876	51.6006	63	4,342	83
21207914	SRR11308075	JAASPP000000000	10/301/209.15	3,158,450	269,879	50.55	4,813,087	135.6285	54	4,516	81
20309619	SRR11308074	JAASPQ000000000	10/301/237.18	1,417,772	218,940	49.99	4,729,242	70.5266	46	4,464	82
20309640	SRR11308072	JAASPS000000000	10/301/229.5	2,580,020	195,665	50.19	4,730,434	124.0743	47	4,469	82
31909860	SRR11308071	JAASPT000000000	10/301/228.5	2,584,654	317,365	50.56	4,791,304	122.4258	40	4,363	77
40810751	SRR11308070	JAASPU000000000	10/301/243.97	1,206,330	240,293	51.15	4,649,880	62.6257	46	4,330	77
21407796	SRR11308068	JAASPW000000000	10/301/226.12	1,105,090	189,899	50.59	4,723,913	52.4501	48	4,389	82
21007859	SRR11308067	JAASPX000000000	10/301/240.83	3,109,500	176,628	50.71	4,531,336	163.8162	53	4,183	83
32309478	SRR11308065	JAASPY000000000	10/301/246.25	1,700,776	217,040	50.62	4,767,857	86.8263	44	4,434	86
10209356	SRR11308062	JAASQB000000000	10/301/230.89	1,159,016	115,051	50.26	4,943,124	53.4247	89	4,678	80
40611716	SRR11308061	JAASQC000000000	10/301/245.98	794,488	157,518	50.64	4,836,525	39.9152	76	4,496	80
10407462	SRR11308060	JAASQD000000000	10/301/235.68	1,678,372	147,706	50.37	4,764,641	81.8932	76	4,430	85
31801812	SRR11308059	JAASQE000000000	10/301/227.22	1,111,140	136,115	50.53	5,134,630	48.2585	108	4,832	83
31501194	SRR11308058	JAASQF000000000	10/301/246.16	2,156,256	119,769	50.2	4,852,335	107.7113	89	4,534	84
21501982	SRR11308057	JAASQG000000000	10/301/193.84	690,546	102,857	50.57	4,553,371	28.7817	85	4,195	78

### Data availability.

These nucleotide sequences have been deposited in DDBJ/ENA/GenBank as BioProject PRJNA612640 under the accession numbers provided in Table 1.
